# Response of Microbial Community Function to Fluctuating Geochemical Conditions within a Legacy Radioactive Waste Trench Environment

**DOI:** 10.1128/AEM.00729-17

**Published:** 2017-08-17

**Authors:** Xabier Vázquez-Campos, Andrew S. Kinsela, Mark W. Bligh, Jennifer J. Harrison, Timothy E. Payne, T. David Waite

**Affiliations:** a UNSW Water Research Centre and School of Civil and Environmental Engineering, The University of New South Wales, Sydney, New South Wales, Australia; b NSW Systems Biology Initiative, School of Biotechnology and Biomolecular Sciences, The University of New South Wales, Sydney, New South Wales, Australia; c Institute for Environmental Research, Australian Nuclear Science and Technology Organisation, Kirrawee DC, New South Wales, Australia; Georgia Institute of Technology

**Keywords:** shotgun metagenomics, radionuclides, plutonium, americium, functional profile

## Abstract

During the 1960s, small quantities of radioactive materials were codisposed with chemical waste at the Little Forest Legacy Site (Sydney, Australia) in 3-meter-deep, unlined trenches. Chemical and microbial analyses, including functional and taxonomic information derived from shotgun metagenomics, were collected across a 6-week period immediately after a prolonged rainfall event to assess the impact of changing water levels upon the microbial ecology and contaminant mobility. Collectively, results demonstrated that oxygen-laden rainwater rapidly altered the redox balance in the trench water, strongly impacting microbial functioning as well as the radiochemistry. Two contaminants of concern, plutonium and americium, were shown to transition from solid-iron-associated species immediately after the initial rainwater pulse to progressively more soluble moieties as reducing conditions were enhanced. Functional metagenomics revealed the potentially important role that the taxonomically diverse microbial community played in this transition. In particular, aerobes dominated in the first day, followed by an increase of facultative anaerobes/denitrifiers at day 4. Toward the mid-end of the sampling period, the functional and taxonomic profiles depicted an anaerobic community distinguished by a higher representation of dissimilatory sulfate reduction and methanogenesis pathways. Our results have important implications to similar near-surface environmental systems in which redox cycling occurs.

**IMPORTANCE** The role of chemical and microbiological factors in mediating the biogeochemistry of groundwaters from trenches used to dispose of radioactive materials during the 1960s is examined in this study. Specifically, chemical and microbial analyses, including functional and taxonomic information derived from shotgun metagenomics, were collected across a 6-week period immediately after a prolonged rainfall event to assess how changing water levels influence microbial ecology and contaminant mobility. Results demonstrate that oxygen-laden rainwater rapidly altered the redox balance in the trench water, strongly impacting microbial functioning as well as the radiochemistry. Two contaminants of concern, plutonium and americium, were shown to transition from solid-iron-associated species immediately after the initial rainwater pulse to progressively more soluble moieties as reducing conditions were enhanced. Functional metagenomics revealed the important role that the taxonomically diverse microbial community played in this transition. Our results have important implications to similar near-surface environmental systems in which redox cycling occurs.

## INTRODUCTION

The rapid expansion of an emerging nuclear industry immediately following World War II resulted in substantial volumes of low-level radioactive waste (LLRW) being generated from nuclear fuel cycle, weapons production, medical radioisotope, and radiochemical research activities. Although there was no consensus at this time, low-level waste (and in some cases more-active material) was commonly disposed of by burial in shallow trenches, as evidenced in the United States at Maxey Flats (1), Oak Ridge (2), and Hanford (3, 4), in Canada at Chalk River (5), in the United Kingdom at Harwell (6) and at an LLRW disposal site (7), in Lithuania at Maišiagala (8), and more recently in Ukraine at Chernobyl (9), to name but a few. This was also the case for Australia's only nuclear (research) reactor at Lucas Heights. Known as the Little Forest Legacy Site (LFLS), radioactive materials, including minor amounts of ^239+240^Pu and ^241^Am were placed in narrow (0.6-m), 3-m-deep, unlined trenches from 1960 to 1968 (10–12). Large volumes of contaminated nonradioactive materials and equipment were also disposed of in these trenches (10).

The LFLS trenches were excavated within undisturbed geological matrices of red-brown and gray clay (primarily kaolinite and illite/smectite) derived from the underlying weathered shale, interspersed with minor phases of hematite and goethite (11, 13). The site was chosen in part due to the low hydraulic conductivities of these materials (∼9 to 66 mm/day) in order to isolate the LLRW from waters associated with the local hydrology cycle (10–12). However, an unintended consequence of this (also experienced at other disposal sites) has been that periodic intense rainfall and prolonged dry conditions can facilitate complete saturation and desaturation of the more-permeable waste material via surface infiltration and evapo(transpi)ration/leakage mechanisms, respectively. Elaborating further, this frequently results in infiltrating water filling up the more-porous trenches to the surface (akin to a “bathtub”), which has been shown to be a primary mechanism for the dispersion of contaminants ^239+240^Pu and ^241^Am (10). Furthermore, this has allowed for redox cycling to occur unabated in the LLRW trenches since their construction, potentially promoting redox-tolerant plasticity in the microbial communities present (14).

With actinide mobility, in many instances, strongly dependent on their oxidation state (15), microbial communities can potentially play decisive roles in determining the fate and mobility of such elements in the environment. In general, microbial communities can influence actinide chemistry by partaking in redox (16–18), dissolution (19, 20), precipitation (21, 22), sorption (23), and/or methylation (24) reactions, which may either enhance or retard contaminant mobility.

Despite this, much of the scientific research to date has focused on isolated individual species (such as those of Geobacter, Shewanella, and Clostridium spp.) and their impact on actinide, particularly uranium, behavior. The challenge remains as to how best to identify the role of microorganisms and their functioning within the wider microbial community which may be undergoing external, environmental changes (25). Furthermore, subsurface environments such as aquifers and shallow groundwaters, aside from being poorly studied, have been shown to be havens for microbial novelty not dominated by the well-characterized organisms listed above (26). Culture-independent techniques, such as metagenomics, in which genomic sequences capture the aggregate microbial ecology of a sample (27, 28) have the potential to enhance our understanding of these complex biogeochemical systems.

The question remains at LFLS, and in similar contaminated redox-cycling environments, as to what function microbial communities may be performing in the direct or indirect mobilization and/or retention of legacy radionuclides. As the establishment of causality between the legacy contaminants and microbial communities cannot be achieved in such a noninvasive environmental study, the aim of this research was to understand the role that periodic inundation from a large rainfall event, and presumably oxygen penetration, had on the concomitant changes to chemistry-radiochemistry and microbial communities in an LLRW trench environment. The two contaminants of major concern, Pu and Am, were the focus of this research, with shotgun metagenomics used to examine the microbial systems function and taxonomy.

## RESULTS AND DISCUSSION

### Water level changes and chemical analyses.

The initial 220-mm rainfall event resulted in trenches filling to capacity and discharging from the surface or porous near-surface (0- to 0.2-m) in the “bathtub” mechanism described by Payne et al. (10) (see Fig. S1 in the supplemental material). Despite the subsequent 47-day sampling period receiving a further 72 mm of rainfall, which periodically increased trench water levels, an overall decline in trench water levels was observed across the sampling period (Fig. S1).

**FIG 1:**
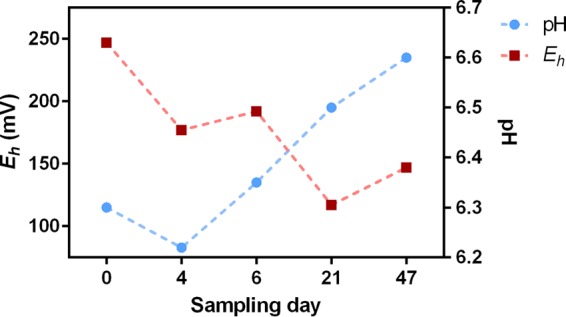
pH and E_h_ (standard hydrogen electrode corrected) measurements from the trench water across the sampling period.

**FIG 2:**
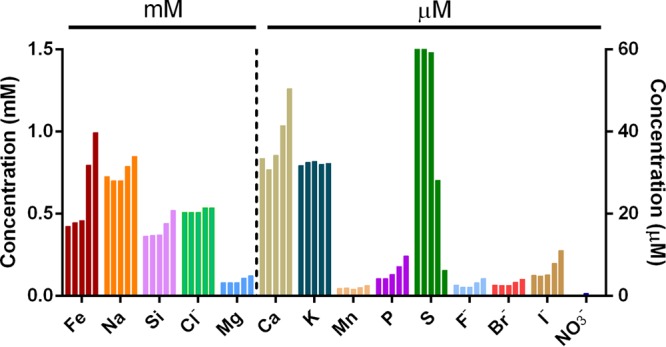
Temporal changes to element/ion concentrations in the trench water. Bars show the individual concentrations of each element/ion over the five sampling days 0, 4, 6, 21, and 47.

Across the sampling period and as the water level declined, the pH was observed to increase from 6.30 to 6.60, whereas the E_h_ values decreased from 247 to 147 mV (Fig. 1). Of the cations and anions analyzed (Fig. 2), iron displayed one of the greatest variations in concentration, increasing from 0.42 mM at day 0 to 1.00 mM by day 47. The elevated concentrations of iron are unsurprising, given that the LLRW at LFLS was disposed of in part within ∼760 steel drums (29) and buried within a highly weathered shale geological matrix (11). The continuous presence of Fe(II), even at day 0, confers reducing conditions in excess of E_h_ values recorded, casting uncertainty over the absolute values supplied by the E_h_ probe. The increase in Fe(II) concentrations with time was likely due to Fe(III) oxyhydroxide reduction, an observation supported by the concurrent liberation of Si and P, two elements that are typically coassociated with Fe(III)-oxyhydroxide at LFLS (30). The constant concentrations of expected conservative elements Cl^−^ and K suggest that the drop in the trench water level was due to subsurface outflow rather than evaporative processes across the course of sampling. Other important elemental transitions included a 10-fold decrease in dissolved sulfur concentrations (presumed to be sulfate), from an initial concentration of 62.4 to 6.2 μM at day 47 (Fig. 2). Nitrate concentrations doubled between days 0 and 6 (from 0.24 to 0.55 μM), after which they decreased to below detection limits at days 21 and 47 (Fig. 2).

**FIG 3:**
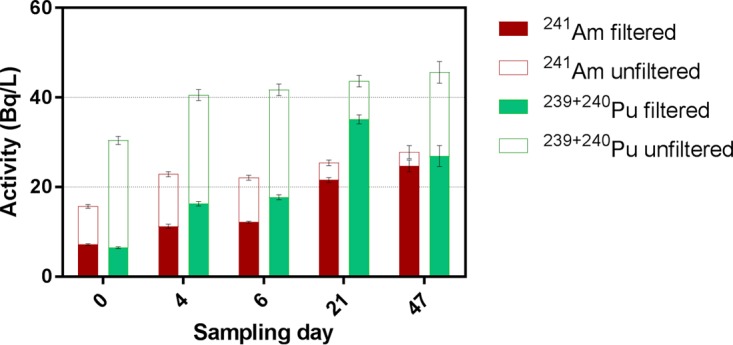
Activity of filtered and unfiltered radionuclides measured in the trench water. Filtered fractions (<0.45 μm, solid fill) are equivalent to soluble and smaller colloidal particles. Unfiltered fractions (full bar) are considered total (soluble plus all suspended solids) concentrations. Error bars show the standard deviations of triplicate measurements.

The total (unfiltered) ^241^Am activity increased from 15.7 to 27.8 Bq/liter (1.8-fold increase), while the soluble (filtered) fraction increased 3.4 times (7.2 to 24.7 Bq/liter) between days 0 and 47 (Fig. 3). Although the activity of the total ^239+240^Pu showed a proportionally smaller increase than ^241^Am across the sampling period, from 30.4 to 45.6 Bq/liter (1.5 times), the soluble fraction increased from 0.21 at day 0 and reached a maximum of 0.80 (35.1 Bq/liter) by day 21. Despite the low-flow sampling conditions, the majority of ^241^Am and ^239+240^Pu in the trench water was solid associated at day 0. This implies that the solid-associated actinides are relatively stable or easily (re)mobilized and also that during the rapid influx of rainwater, when the trenches are filling up (and potentially overspilling), colloidal transport is likely to be the major form of contaminant movement at this site. Previous redox state measurements at LFLS found that Pu was present in the Pu(IV) (64.5%) and colloidal/Pu(III) (35.5%) states while Am was present exclusively as Am(III) (31). The vast quantities of Fe(II) as well as the results of ancillary measurements (dissolved oxygen [DO], oxidation/reduction potential [ORP]) would suggest that Pu(IV) and Am(III) were again the dominant oxidation states of the trench water contaminants. The results of all water quality parameters measured are provided in Table 1.

**TABLE 1:** Geochemical parameters measured in the trench water

Parameter	Value at sampling day (date)^*a*^					Maximum background concn^*b*^
	0 (23 April)	4 (27 April)	6 (29 April)	21 (14 May)	47 (9 June)	
Field parameters						
pH	6.3	6.22	6.35	6.5	6.6	NA
E_h_ (mV)^*c*^	247	177	192	117	147	NA
DO (mg/liter)	0.5	0.6	0.6	0.6	0.5	NA
TDS^*h*^ (g/liter)	0.115	0.12	0.118	0.156	0.185	NA
Temp (°C)	20.9	19.5	19.8	18.2	19.2	NA
Water level (m)^*d*^	−0.89	−1.26	−1.36	−1.55	−1.64	NA
Radiochemistry (activity in Bq/liter)^*e*^						
^241^Am (UF)^*f*^	15.68 ± 0.38	22.85 ± 0.58	22.09 ± 0.55	25.4 ± 0.64	27.8 ± 1.49	NA
^241^Am (F)^*g*^	7.17 ± 0.19	11.24 ± 0.48	12.15 ± 0.22	21.56 ± 0.59	24.71 ± 1.3	NA
^239+240^Pu (UF)^*f*^	30.44 ± 0.9	40.53 ± 1.22	41.71 ± 1.29	43.66 ± 1.27	45.6 ± 2.43	NA
^239+240^Pu (F)^*g*^	6.46 ± 0.21	16.28 ± 0.51	17.73 ± 0.56	35.11 ± 0.99	26.93 ± 2.36	NA
Chemistry						
DOC (mg/liter)	4.07	4.15	5.30	5.91	6.77	0.04
Fe(II) (mM)	0.34	0.39	0.45	0.75	0.92	<0.01
Fe (mM)	0.42	0.45	0.46	0.80	0.99	<0.01
Na (mM)	0.73	0.70	0.70	0.79	0.85	<0.01
SiO_2_ (mM)	0.36	0.37	0.37	0.44	0.52	<0.01
Cl^−^ (mM)	0.51	0.51	0.51	0.54	0.54	0.01
Mg (mM)	0.08	0.08	0.08	0.11	0.12	<0.01
Ca (μM)	33.43	30.69	34.18	41.42	50.40	0.25
K (μM)	31.71	32.48	32.74	31.97	32.23	0.31
Mn (μM)	1.80	1.86	1.64	2.00	2.55	<0.02
P (μM)	4.20	4.20	5.17	7.10	9.69	<1.6
S (μM)	62.36	62.36	59.25	28.06	6.24	<3.11
F^−^ (μM)	2.63	2.11	2.11	3.16	4.21	<0.53
Br^−^ (μM)	2.63	2.50	2.50	3.25	4.01	<0.13
I^−^ (μM)	4.96	4.81	5.12	7.88	11.03	<0.22
NO_3_^−^ (μM)	0.24	0.24	0.55	0.00	0.00	<0.01

a Day count from the first day with no precipitation after the rainfall event. All dates are for the year 2015.

b Highest concentrations measured on multiple blanks (*n* > 5) processed in parallel with trench samples. NA, not applicable.

c Corrected values against standard hydrogen electrode.

d Depth below ground surface.

e Plus or minus 1 standard deviation.

f Filtered through 0.45-μm filter (soluble/colloidal).

g Unfiltered (total).

h TDS, total dissolved solids.

### Community composition.

Community profiles showed that Bacteria dominated over Archaea in the trench water across the entire sampling period. A total of 70 phyla of the 85 included in the GreenGenes database were detected at some point, and 11 phyla with values of >1% of the total community were detected at all times. Eukarya were found in low (∼1% to ∼2%), nearly constant abundance throughout the samples. Similarly, eukaryotic-specific family groups determined by MetaCyc reactions (RXNs) were all below our threshold significance level. As such, they are not considered in further detail or for the taxon abundance numbers throughout this paper.

**FIG 4:**
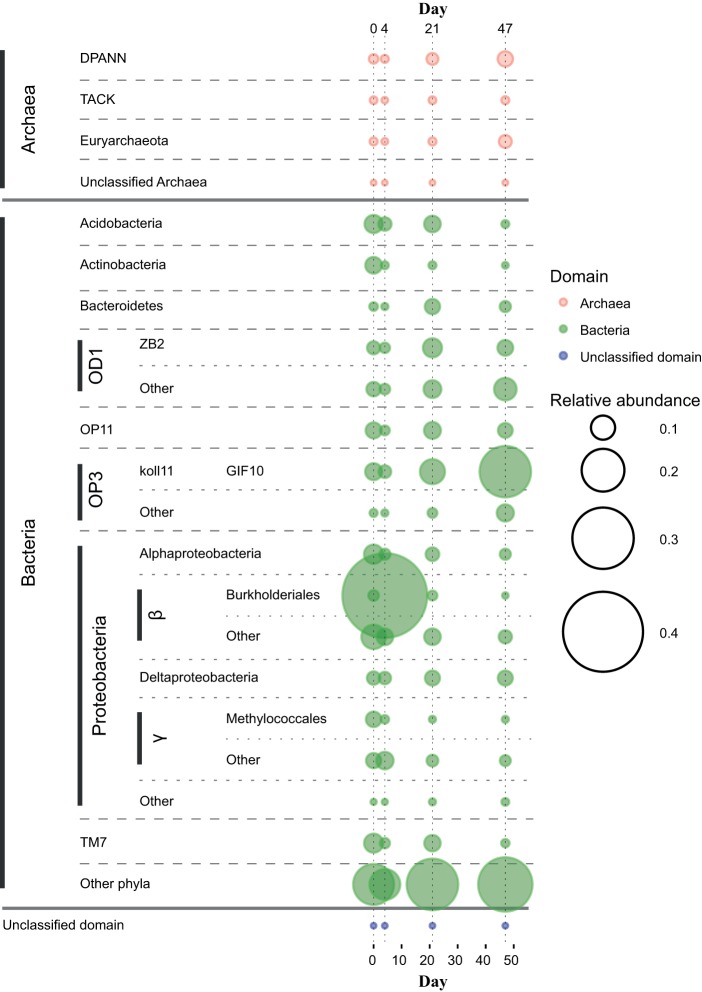
Taxon relative abundances over time. Only taxa with an average relative abundance of >5% of the total sequences in at least a single sampling day are shown. Taxa with <5% of all sequences are grouped under “other.” Phyla with <5% of relative abundance at all times are grouped under “other phyla,” except Archaea.

Archaea oscillated between 2.6 and 10.6% of the classified reads with minimum and maximum at days 4 and 47, respectively. Micrarchaeota and Parvarchaeota (superphylum DPANN) were the most abundant phyla at all times with a combined 45.5 to 55.9% of Archaea, while remaining sequences were shared in variable proportions between the superphylum TACK and Euryarchaeota (Fig. 4). Although the fraction of TACK decreased over time from 24.8% at day 0 to 12.4% at day 47, Euryarchaeota reached a maximum at day 47, contributing 37.2% of all Archaea. These changes were derived mainly from variations in the SAGMA-X family (Thaumarchaeota), related to the ammonia-oxidizing archaeon “Candidatus Nitrosotalea” (32, 33), and in Bathyarchaeota (Miscellaneous Crenarchaeotal Group [MCG]), which includes the only potential noneuryarchaeal methanogens (33, 34) and/or one of the few acetogenic Archaea (35). The increase in total abundance of Archaea over time was related to changes in ANME-2d Methanoregulaceae and Methanosaetaceae families, implicated in methane metabolism (36–38), as well as the Micrarchaeota and Parvarchaeota phyla.

Only 8 bacterial phyla grouped more than 5% of the total bacterial reads at any one sampling point, and among them, only Chloroflexi, OD1 (Parcubacteria), OP11 (Microgenomates), OP3 (Omnitrophica), and Proteobacteria were within the 10 most abundant bacterial phyla at all times (Fig. 4). The principal changes observed over time refer to Proteobacteria, TM7 (Saccharibacteria), OP3, Acidobacteria, and OD1. While the proportion of TM7 and Acidobacteria decreased from day 0, both OP3 and OD1 reached their maximum by day 47 (18.9% and 14.7%). The Proteobacteria, often the most abundant phylum, showed a conspicuous increase, mainly due to Betaproteobacteria, comprising 48.7% of all classified reads at day 4. The order Burkholderiales was identified as being primarily responsible for this change (42.6% of total), especially the genus Ralstonia (20.3%) within Oxalobacteraceae (34.0%). These bacteria were also responsible for the altered functional profile observed at day 4.

Several studies focusing on the microbiology of groundwater or subsurface ecosystems have demonstrated the existence of organisms able to pass through standard 0.22-μm filters (39–41). Despite this, a substantial portion of the Little Forest trench water community corresponded to taxa known to be able to pass through 0.22-μm filters, i.e., OD1, OP11, and DPANN. We suggest that this could be derived from the fast precipitation of iron in our extracted trench waters, effectively entrapping cells, or that they were attached to colloidal particles or to other cells. Still, it is possible that the numbers for these “ultrasmall” taxa could be underestimated in our results.

A complete taxonomic profile of all sample replicates at each individual time point is provided in the supplemental material. An important aside to be noted with these data is the inherent reproducibility between sample triplicates. This finding provides a level of reassurance with regard to the aggregate nature of these water samples, often neglected in similar studies.

### Carbon cycling.

**FIG 5:**
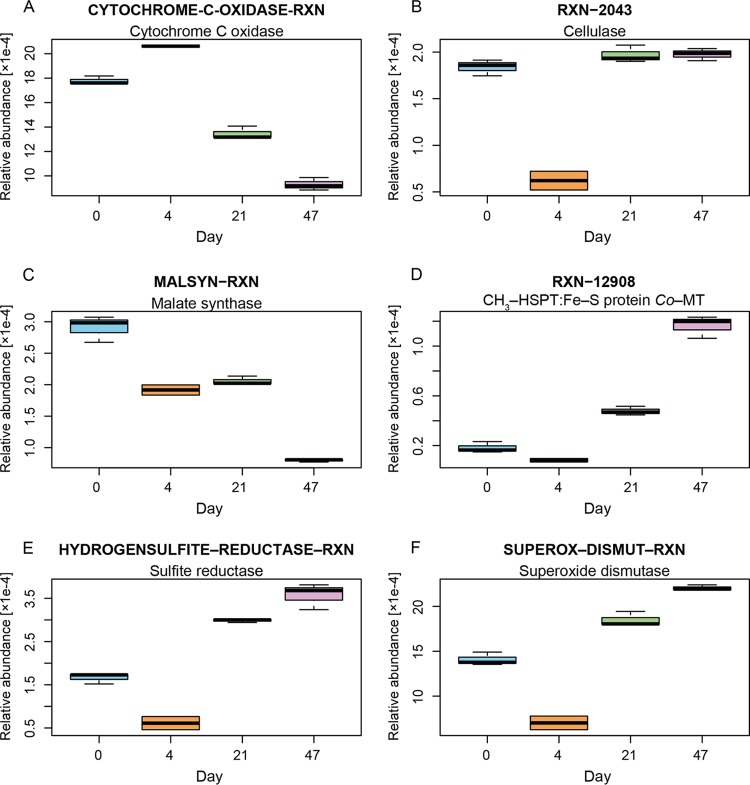
Changes in the relative abundances of selected RXNs over time. (A) Cytochrome *c* oxidase; (B) cellulase; (C) malate synthase; (D) 5-methyltetrahydrosarcinapterin:corrinoid/iron-sulfur protein co-methyltransferase (CH3-HSPT:Fe-S protein Co-MT); (E) sulfite reductase; (F) superoxide dismutase.

Initial time points were characterized by a significantly (*P* ≤ 0.05) higher relative abundance of RXNs using molecular dioxygen as the substrate, such as cytochrome *c* oxidase (CYTOCHROME-C-OXIDASE-RXN, EC 1.9.3.1 [Fig. 5A]) (MetaCyc RXN notations are shown here in all-uppercase format to provide an exact match with the MetaCyc database), quinol-cytochrome *c* reductase (1.10.2.2-RXN), and several mono- and dioxygenases. This was combined with a significantly (*P* ≤ 0.05) lower relative abundance of methanogenesis-related RXNs, nitrogenase (ferredoxin, NITROGENASE-RXN), rubisco (RIBULOSE-BISPHOSPHATE-CARBOXYLASE-RXN), or heterolactic fermentation (PHOSPHOKETOLASE-RXN). The catalase (CATAL-RXN, EC 1.11.1.6/21 [see “Reactive oxygen species detoxification” in the supplemental material]) gene's relative abundance was highest at day 4 along with those of several ABC transporters and phosphotransferases, e.g., d-ribopyranose, d-xylose, spermidine, or l-arginine, as well as assimilatory sulfur and nitrogen pathway RXNs (refer to the supplemental material for details). The higher relative abundances of all these genes at day 4 suggest a metabolism more dependent on oxygen, i.e., aerobic, or at least microaerophilic (see “Nitrogen cycling” below). It also confers a higher dependence on available organic carbon (*viz*., heterotrophy) or on the presence of decaying complex organic matter (42). These interpretations are collectively supported by the geochemical measurements, including the higher E_h_ values, decreasing concentrations of sulfur, and absence of nitrate, along with lower iron concentrations across the first sampling points.

Regarding the source of the organic fraction, the results suggest that soil particles and associated organochemicals were mobilized from material above the trenches via advective transport mechanisms during and immediately after rainfall. This was evidenced by the increased proportion of soil-associated Actinobacteria at day 0, which diminished to 0.71% by day 4 (Fig. 4). Furthermore, the presence of genes encoding enzymes representing chitin degradation, such as chitobiase (RXN-12625, EC 3.2.1.52), diminished progressively until day 47, indicating a potential one-off provision of chitin, matching the leaching hypothesis (see Fig. S2 in the supplemental material). In addition, numerous facultative and strict anaerobes can degrade chitin and/or chitobiose (43–45), which lessens the likelihood of the alternative hypothesis that chitin depletion could be derived from the intrinsic lack of chitobiase in anaerobes.

The other primary biopolymer found in soil is cellulose (46). The relative abundance of cellulase (RXN-2043, EC 3.2.1.4) showed no significant differences between days 0, 21, and 47 (*P* = 0.172), suggesting a consistent supply of cellulose (Fig. 5B). The exception, noted at day 4, can be explained by the proliferation of Burkholderiales. The constant abundance of cellulose is unsurprising given the historical records, which indicate that a range of organic (particularly cellulose-based) compounds were codisposed of in the LFLS trenches (11). This hypothesis accords with previous stable isotope measurements, which identified the degradation of legacy organic matter as being responsible for enriched δ^13^C (inorganic) values measured near the trench (11).

Lignin is present to a lesser or greater extent in all plant biomass. From the list of enzymes in the literature capable of degrading lignin (47, 48), only below-threshold levels of certain genes were detected: versatile peroxidase (RXN0-267, EC 1.11.1.16), dye peroxidase (RXN-11813, EC 1.11.1.19), and laccase (LACCASE-RXN, EC 1.10.3.2). The overall lack of genes for proteins capable of lignin degradation strengthens the hypothesis that trench waste material is the source of cellulosic material rather than being derived from the degradation of plant material from the topsoil.

The two fundamental genes representing the glyoxylate pathway, isocitrate lyase (ISOCIT-CLEAV-RXN, EC 4.1.3.1) (see Fig. S3 in the supplemental material) and malate synthase (MALSYN-RXN, EC 4.1.3.2) (Fig. 5C) showed between 3 and 4 times higher relative abundance on days 0 and 4 than on day 47. This pathway facilitates the use of short-chain carbon compounds (C_2_ or C_3_) for anabolic purposes, compounds especially generated during anaerobic processes. Although the glyoxylate pathway genes have been previously detected in organisms living in anaerobic environments (49), they were absent from strict anaerobic organisms based on the results from EggNOG (50), KEGG (51), and UniProtKB (52) databases (as of 24 May 2017). This finding supports the hypothesis that trench waters became anoxic as the water level declined, promoting conditions that favor the development of strict anaerobes. Furthermore, the aerobic community that developed immediately after the rainfall event likely utilized (and benefited from) the short-chain carbon compounds generated by preceding anaerobic conditions.

Both acetoclastic and hydrogenotrophic methanogenesis pathways were evident from the functional profile analysis. Similar maximum relative abundances, about 1.4 × 10^−4^, of 5-methyltetrahydrosarcinapterin:corrinoid/iron-sulfur protein co-methyltransferase (RXN-12908, EC 2.1.1.245) (Fig. 5D) and coenzyme F_420_-dependent hydrogenase (COENZYME-F420-HYDROGENASE-RXN, EC 1.12.98.1) (see Fig. S4 in the supplemental material), which are specific RXNs for the methanogenesis from acetate and H_2_ and CO_2_, were observed at day 47. The presence of methanogenesis-specific RXNs is supported by previous isotopic measurements, showing δ^2^H enrichment, relative to δ^18^O, in the vicinity of the trenches (11). Concurrently, the taxonomy revealed the presence of the ANME-2d division contributing up to 13.8% of all Archaea at day 47 (1.5% of the community). The ANME-2d division was initially linked to the anaerobic oxidation of methane using NO_3_^−^ (36) or even NO_2_^−^ (53), constituting one of the primary methane sinks under anaerobic conditions. However, recent research has shown that some of the members of this taxon are able to utilize Fe(III) in place of NO_2_^−^ or NO_3_^−^ (38, 54, 55). This provides a more reasonable explanation given the limited nitrate present and abundance of iron during the later sampling days.

### Sulfur cycling.

The relative abundance of sulfite cytochrome *c* reductase (SULFITE-DEHYDROGENASE-RXN, EC 1.8.2.1) (see Fig. S5 in the supplemental material), involved in the assimilatory reduction of sulfate, peaked at day 4, with ∼10 times higher relative abundance than on day 0. At the same time, dissimilatory sulfate reduction RXNs, e.g., dissimilatory sulfite reductase (HYDROGENSULFITE-REDUCTASE-RXN, EC 1.8.99.3), followed the opposite trend, with a maximum at day 47 (Fig. 5E). The combined relative abundance of all the putative sulfate-reducing bacterium (SRB) taxa detected (mainly Syntrophobacterales, Thermodesulfovibrionaceae, and Desulfobacterales) also increased over time, from 2.3% of the total prokaryotes at day 0 to 4.9% at day 47. Dissimilatory sulfate reduction is one of the major redox processes in both natural (56) and artificial (57) anaerobic environments. The 10-fold reduction in the sulfur concentration is thought to result from the loss of dissolved sulfate via reduction to sulfide and subsequent precipitation of insoluble heavy metal sulfides, particularly FeS. This interpretation is supported by the lack of measurable sulfides in the trench water (see “Chemical analyses of trench waters” below), along with previous reports showing sulfate reduction based on isotopic fractionation and severe (10- to 100-fold) depletion in sulfate concentrations in trench waters relative to surrounding wells (11). The potential contribution of sulfate- and nitrite-dependent anaerobic methane oxidation (S-DAMO and N-DAMO, respectively) was discounted due to the near-complete absence of ANME taxa (Archaea) aside from ANME-2d, and NC10 (Bacteria) (see “Carbon cycling” above) (38, 53, 58, 59).

### Nitrogen cycling.

The (inorganic) nitrogen cycle was represented mostly by RXNs related to denitrification and assimilatory nitrogen reactions, primarily dissimilatory nitrate reductase (RXN-16471, EC 1.7.5.1) (see Fig. S6 for a complete profile of the N-associated RXNs), although nitrogen fixation (NITROGENASE-RXN, EC 1.18.6.1) was the predominant N-associated RXN. All other prominent N-associated RXNs peaked at day 4 with the exception of hydroxylamine reductase (HYDROXYLAMINE-REDUCTASE-RXN, EC 1.7.99.1) and ammonia oxygenase (AMONITRO-RXN, EC 1.14.99.39). The increase in nitrate concentrations observed between days 4 and 6 (0.236 and 0.552 μM, respectively) might be derived from the oxidation of NO by nitric oxide dioxygenase (R621-RXN, EC 1.14.12.17). This surge in the relative abundance of nitrogen metabolism RXNs (at day 4) coincided with an increased dominance of Betaproteobacteria, mostly Burkholderiales. Betaproteobacteria have been linked to denitrification processes in a uranium-polluted aquifer in Rifle, CO, USA (60), and they may play a similar ecological role at LFLS. However, the increase in nitrate concentrations could equally be indicative of NO or NO_2_^−^ oxidation, originally produced by the aerobic oxidation of ammonium by the Thaumarchaeota (61, 62).

The inorganic nitrogen cycling results provide important information regarding the transition from aerobic to anaerobic conditions as the water level declines. The competing requirements of nitrate respiration, which necessitates oxygen-limiting conditions at a minimum (63), and NO dioxygenase, needing molecular oxygen, collectively suggest that at day 4 the trench waters were no longer aerobic but more likely microaerophilic/hypoxic. However, many facultative anaerobes are capable of using nitrate as an alternate electron acceptor when oxygen is not available. As such, the high relative abundance of nitrate reducing enzyme genes at day 4 (*viz*., dissimilatory nitrate reductases) could be a confounding effect associated with the increased abundance of Burkholderiales. This was confirmed by searching the denitrifying pathway in KEGG (map00910) (as of 8 July 2016) (51).

### Iron cycling.

Temporal increases in Fe(II) concentrations, such as those experienced in the later anoxic periods of the sampling, may be associated with the use of Fe(III) during anaerobic respiration, previously attributed to decaheme *c*-type cytochromes from the OmcA/MtrC family present in iron-reducing bacteria (FeRB) such as Shewanella spp. and Geobacter spp. (64). However, their representation in the trench water metagenome data was scarce (relative abundance, ≤10^−6^). It is known that for respiration to occur, FeRB often require direct physical contact with Fe(III) solids (65). As only aqueous/suspended-phase sampling was permissible at LFLS, it is possible that the underrepresentation of an FeRB community was due to our sampling regime in which the solid phase was largely excluded.

The genes associated with RXNs specific to the Fe(II) oxidation pathway (via rusticyanin, PWY-6692), i.e., ubiquinone-cytochrome-*bc1* reductase (EC 1.10.2.2, RXN-15829) and *aa3*-type cytochrome oxidase (EC 1.9.3.1, RXN-15830), were present, despite no iron-rusticyanin reductase (EC 1.16.9.1, RXN-12075), *cyc2*, being found under the established threshold (maximum value, ∼3 × 10^−6^) (66). The presence of both cytochromes may be explained by the fact that HUMAnN2 could not distinguish between the “generic” quinol-cytochrome *c* reductase (RXN-15816, EC 1.10.2.2) and cytochrome *c* oxidase (CYTOCHROME-C-OXIDASE-RXN), particularly when analyzing short reads.

The presence of 1.9% of Gallionellaceae (over the total community composition) at day 0 and that of 2.9% of Crenothrix, a non-iron-oxidizing bacterium (non-FeOB) usually associated with Fe-mineralizing biofilms (67), suggests the existence of transient microaerophilic iron-oxidizing activity in the trench water despite the extremely low relative abundance of *cyc2*. Waste disposal records from LFLS indicate that numerous steel drums (∼760) were codisposed of in the trenches (29). Based on the active sulfate respiration observed in the trench waters, we would expect that iron/steel materials may have suffered microbially induced corrosion (MIC) (68), potentially contributing to the elevated Fe(II) concentrations. However, the main iron MIC product is likely FeS (68), which can be utilized by Gallionella ferruginea (Gallionellaceae), an FeOB (69). Furthermore, this process would also provide an explanation for a persistent source of Fe(III) (oxy)hydroxides and their ongoing interaction with the S cycle.

Previous research on soils experiencing fluctuating redox conditions and active iron cycling has shown that they frequently lack “typical” Fe(III)-respiring bacteria (70), because these organisms are outcompeted by sulfate respiration (71). Despite Fe(III) being a more thermodynamically favorable electron acceptor, it is commonly acknowledged that sulfate respiration often outcompetes Fe(III) respiration in both high- and low-sulfate environments (71, 72). High levels of sulfate reduction in low-sulfate environments, akin to the LFLS trenches, has been previously observed to occur in the presence of crystalline Fe(III) oxyhydroxides that partially reoxidize sulfide generated by SRB to elemental sulfur (72). This mechanism provides a plausible explanation for our observations at LFLS. The large concentration of dissolved Fe(II) in the trench waters would likely contribute to the Fe(II)-catalyzed transformation of amorphous ferrihydrite to more crystalline, thermodynamically favorable forms (73, 74), although this would be limited to some extent by the large concentrations of dissolved organic matter and silica (75). Furthermore, the abiotic reduction of crystalline Fe(III) oxyhydroxides would severely limit the energy acquisition by FeRB, and therefore, the FeRB would only outcompete SRB when sulfate and other reducible sulfur compounds were totally consumed.

Alternatively, fermentative bacteria coupling the oxidation of a range of organic compounds to the reduction of ferric iron (76–78) may play an integral role in maintaining the elevated Fe(II) concentrations observed within the trenches. One example is the organism Propionibacterium freudenreichii, which has previously been observed to reduce Fe(III) by using humic substances as an electron mediator (79). In this regard, both heterolactic and propionic acid fermentation pathways showed high relative abundances based on phosphoketolase (PHOSPHOKETOLASE-RXN, EC 4.1.2.9) and methylmalonyl-coenzyme A (methylmalonyl-CoA) decarboxylase (RXN0-310, EC 4.1.1.41), respectively (see Fig. S7 in the supplemental material). Their decrease in relative abundance (at day 4) could be explained by the lack of fermentative pathways present in the genomes associated with the population shift, i.e., increase in Burkholderiales. This was confirmed by searching (as of 8 June 2016) methylmalonyl-CoA decarboxylase and phosphoketolase in the KEGG maps (map00640 and map00030, respectively).

### Synthesis of trench processes and radiochemical mobilization implications.

**FIG 6:**
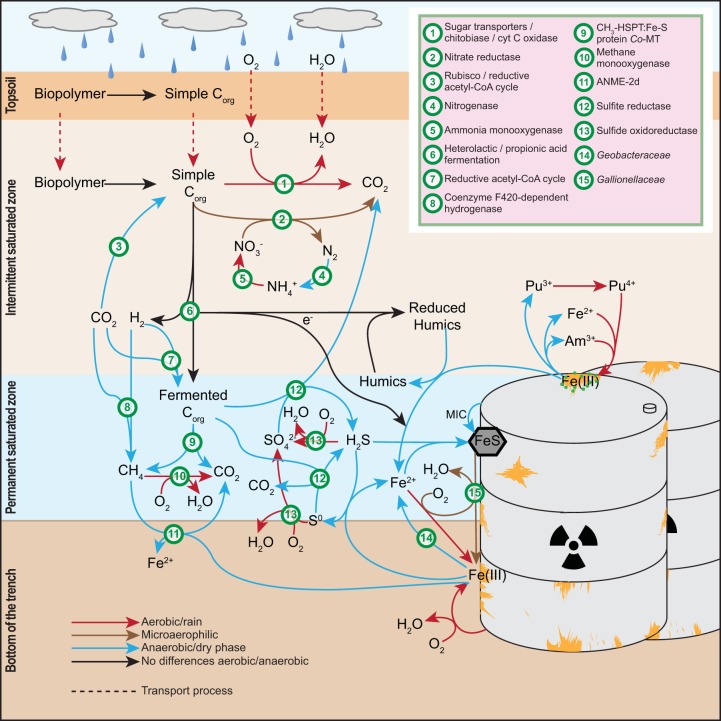
Hypothetical global scheme of the processes at LFLS. Orange details on barrels reading Fe(III) represent solid Fe(III) minerals, mainly (oxy)hydroxides. Colors of arrows represent the time at which each process takes place: red, aerobic/after rain; brown, microaerophilic; blue, anaerobic/dry phase; black arrows indicate processes that seem to be independent from the sampling time. Dashed arrows indicate transport. Abbreviations: C_org_, organic carbon; MIC, microbially induced corrosion; CH_3_-HSPT:Fe-S protein *Co*-MT, 5-methyltetrahydrosarcinapterin:corrinoid/iron-sulfur protein co-methyltransferase.

The biogeochemistry of this dynamic system is conceptually described by the elemental cycling schematic shown in Fig. 6. Immediately following the rainfall event, the E_h_ reached its most-oxidizing value (247 mV) with the microbial community characterized by higher relative abundances of pathways related to aerobic (or at least microaerophilic) heterotrophic metabolism and one capable of chitin degradation. Although conditions were not strongly oxidizing, this pulse of oxic water would be sufficient to induce the abiotic oxidation of part of the large store of dissolved Fe(II), likely forming a combination of the reactive Fe(III) oxyhydroxides ferrihydrite and silica-ferrihydrite, along with the more-crystalline oxyhydroxide lepidocrocite, as has previously been observed in these trench waters (30). As both Am(III) and Pu(III)/(IV) are known to strongly sorb to sediments and iron oxides (15), it is of little surprise to observe the greatest proportions of (suspended) solid-associated actinides at this time point (day 0) (Fig. 3). Note that even though most Am (54.3%) and particularly Pu (78.8%) were associated with a solid fraction of >0.45 μm, they were still extracted from the trench under our low-flow sampling method. This finding implies that colloid-associated Pu, and to a lesser extent Am, remains mobile within the trench waters, congruent with observations from other legacy radioactive waste sites (80). Interestingly though, the Pu migration distance away from source at LFLS in groundwater has been shown to be much smaller (∼1 order of magnitude less) than in other legacy locations such as the Nevada Test Site (81), Rocky Flats (82), and Mayak (83). We attribute this to a low-permeability soil matrix, inhibiting downward migration to the connected/permanent water table, coupled with the biogeochemical conditions within the trenches themselves. The high concentrations of Fe(II), circumneutral pH, and proliferation of aerobic heterotrophs drive the rapid formation of large quantities of Fe(III) oxides. The quantity of Fe(III) oxides that form upon oxic rainwater intrusion is evidently sufficient to contain the bulk of contaminants within the trenches during “bathtub” overflow events. The rate of Fe(II) oxidation is likely to be crucial for the ongoing attenuation of Pu and Am at LFLS (30).

By day 4, as water levels rapidly decrease, an increase of Burkholderiales (Betaproteobacteria) generates a functional profile disturbance, and while the aerobic profile is maintained, it is also the time point at which nitrogen cycling (e.g., nitrate respiration) becomes most active (Fig. 6, green circle number 2). Therefore, day 4 represents a potential transition away from oxic conditions derived from rain infiltration, though this sequence of events is somewhat confounded by the microbial growth lag phase associated with aerobic respirators.

Over the following weeks (days 21 and 47), the microbial community transitions to a functional profile dominated by carbon fixation, methanogenesis, and sulfate respiration pathways (Fig. 6, green circles 3, 8, 9, and 12). The increase in anaerobicity correlated with increasing concentrations of soluble Fe and soluble radionuclides and a depletion of sulfate and nitrate. In the case of Am, although the total activity increased gradually as the water level in the trench declined, the soluble fraction increased by a greater proportion (Fig. 3). This indicates that either a desorption or a dissolution process occurred. The concomitant increases in the soluble Am fraction and Fe(II) concentrations point toward reductive dissolution of Fe(III) oxyhydroxides as a major driver behind Am solubilization.

The Pu behavior in the trenches presents a more-complex temporal dynamic due, in part, to its more-varied redox chemistry. As conditions become more reducing over time, one would expect to observe soluble Pu activities increase, both through the dissolution of Fe(III) oxides and, to a lesser extent, from Pu(IV) to Pu(III) reduction. In an interesting point of difference from Am, our results show that Pu proportionally remained in the particle-associated fraction for a substantially longer period, even as the dissolved Am(III) activity and Fe(II) concentration increased. The reason for the differing solution/solid-phase partitioning observed for Pu and Am is not clear based on the evidence at hand. The inability to sample solid materials from within the trenches has limited more-conclusive understanding. However, we suggest that the differing Am and Pu associations are likely due to different redox states, based on the previous measurements at LFLS, which showed a dominant Pu oxidation state of +4 (31). Electrostatically, Pu(IV), as the neutral hydrolyzed cation Pu(OH)_4_(aq) or as an intrinsic colloid, is more likely sorbed or incorporated into positively charged pseudocolloids such as Fe(III) oxides than Am^3+^. This is supported by research showing the highly reversible nature of Am(III) sorption onto poor crystalline iron colloids (84) and association with carbonate- and exchangeable sites on clays (85).

### Conclusions.

The inability to comprehensively access and sample within legacy radioactive waste environments hampers our ability to comprehend cooccurring elemental cycling and microbial metabolism, potentially curtailing our ability to effectively manage and remediate such sites. The trench-sampling point at LFLS is therefore a particularly useful resource for such research. In this study, our coupled use of metagenomics and chemical analyses has provided a previously unattainable level of understanding for the LFLS trench water, highlighting the responsiveness of the microbial community to external changes and dynamic nature of the resulting chemistry. The combined results show that the trench waters contain a taxonomically diverse microbial community, which has likely evolved in response to variations in energy sources supplied by frequent redox fluctuations. When combined with the complex nature of the waste form, a myriad of microenvironments have developed within the trenches, allowing for simultaneous O, N, Fe, S, and C elemental cycling, as shown by cooccurring metabolic reactions in the aggregate water samples. Consequently, it can be inferred that Pu and Am are subject to persistent reducing conditions (as evident from active iron oxide dissolution, sulfate reduction, and methanogenesis) when the water level is low between rainfall events. These reductive processes maintain Pu and Am solubility, despite the occasional onset of oxidizing conditions associated with rainfall events. Ultimately however, the high concentrations of Fe present and the tendency of Fe(II) to be relatively rapidly oxidized to strongly sorbing Fe(III) (oxy)hydroxide solids on exposure to oxic conditions result in limited transport of Pu and Am.

Although the findings described above are intrinsically linked to the specific site under investigation, they provide important generic insights into the dynamic biogeochemical behavior of iron-rich, redox-cycling environments. Of particular interest is the rapid response of the microbial community to dynamic redox conditions and the potential impact upon persistent contaminant solubility and enhanced mobility.

## MATERIALS AND METHODS

### Sample collection.

Trench water samples were collected on five separate occasions across a 2-month period from LFLS. They were obtained from a screened polyvinyl chloride (PVC) pipe that extends 1.55 m below the ground surface. This pipe provides the only point of access into the legacy trenches and was opportunistically installed during the partial collapse of the trench surface. Further details of the trench sampler have been previously described in the literature (10, 31). Briefly, a peristaltic pump operating under a low flow rate was used to purge the borehole until chemical parameters, particularly pH and oxidation/reduction potential (ORP), became stable (see the supplemental material). Measurements were made using a multiprobe system (YSI 556 MPS) coupled to a 100-ml flow cell. Stability usually took between 30 and 60 min, after which sample collection began. Chemical parameters, along with the water depth, were monitored over the sampling period (typically some hours) on the day of sampling to ensure that the water was representative of that particular period. The stability of flow cell parameters and the fact that the water level did not decrease during the collection of 4 to 5 liters of sample material provide assurance that the samples were representative of the particular event rather than anomalous local conditions.

The sample collection period commenced (on 23 April 2015) immediately after a substantial rainfall event, during which ∼220 mm of rain fell over 3 days (Fig. S1). Trench water samples were subsequently collected on 27 and 29 April, 14 May, and 9 June 2015 (or days 0, 4, 6, 21, and 47 after the rain event) (Fig. S1).

Samples for chemical analyses were collected both with and without an in-line filtration membrane (0.45-μm polyethersulfone [PES], Waterra FHT-Groundwater) in prewashed and trench water-rinsed high-density polyethylene (HDPE) bottles, except samples for organic carbon, which were collected in glass bottles. Samples for radiochemical and cation analyses were acidified using double-distilled HNO_3_. All samples were then stored at 4°C until analysis.

Samples for microbial analyses were collected by directly filtering the trench water using a sterile PES 0.22-μm syringe filter (Sterivex-GP; Merck Millipore) with the volumes of filtrate passing through the membranes recorded (410.1 ± 82.2 ml [mean ± SD] per replicate). Filters were capped on site before being stored at −18°C until analysis. Microbial sampling was performed in triplicate to assess inherent variation.

Due to regulatory protocols governing sampling at the site, no solid-phase material could be extracted from the trenches. As the surrounding geological materials into which the trenches were excavated provide little resemblance both physically and chemically to the deposited waste within the trenches, they were discarded as providing no useful information for this study. As such, all sampling within the trenches pertains to the extraction of soluble or suspended solid phases from the screened borehole.

### Chemical analyses of trench waters.

The primary radiochemical contaminants, ^239+240^Pu and ^241^Am, were separated from other radiochemical fractions using TEVA and TRU resin cartridges (86) before measuring activity by alpha spectroscopy using a Canberra Alpha Analyst system coupled with passive implanted planar silicon detectors (Canberra).

Cations (including Si, S, and P) were measured by either inductively coupled plasma atomic emission spectroscopy (ICP-AES) or ICP mass spectrometry (ICP-MS) depending on relative concentration. Anions (F^−^, Cl^−^, Br^−^, I^−^, PO_4_^3−^, and NO_3_^−^) were determined by ion chromatography (Dionex DX-600 IC System).

Nonpurgeable dissolved organic carbon (0.45 μm filtered) was measured on freshly collected replicate samples by combustion catalytic oxidation (TOC-5000A; Shimadzu) with the concentration determined by a five-point calibration using newly prepared potassium hydrogen phthalate solutions.

Ferrous iron concentrations from 0.45-μm-filtered samples were preserved in the field with ammonium acetate-buffered phenanthroline solution (87). The absorbance of Fe(II) was measured at 510 nm (USB4000; Ocean Optics) calibrated against a freshly prepared ammonium ferrous sulfate solution.

In-field measurements of dissolved sulfide were attempted using methylene blue colorimetry, but any dissolved sulfide was consistently below detection limits (∼1 μM) at all sampling time points.

All chemical analyses were performed in triplicate.

### DNA extraction and sequencing.

The PowerLyzer PowerSoil DNA isolation kit (Mo Bio) was used to extract DNA from Sterivex filters according to the modified protocol of Jacobs et al. (88). Bead beating was performed singularly in a PowerLyzer 24 Bench Top Bead-Based Homogenizer (Mo Bio) at 2,000 rpm for 5 min.

DNA concentration and quality were evaluated with LabChip GX (PerkinElmer). Libraries were prepared with the TruSeq Nano DNA Library Preparation kit and sequenced in a NextSeq 500 (Illumina) with a 2 × 150-bp high-output run. Output consisted of paired-end reads with a median insert size of ∼480 nucleotides (nt).

### Data processing and functional analyses.

Raw sequencing reads were preprocessed with Trim Galore! (http://www.bioinformatics.babraham.ac.uk/projects/trim_galore/) to remove residual adapters as well as short and low-quality sequences (Phred33 ≥ 20) while keeping unpaired reads.

Taxonomic profiles were obtained from shotgun metagenomic data by extracting 16S rRNA gene sequences with GraftM v0.10.1 (https://github.com/geronimp/graftM) and mapping them with the 97% clustered GreenGenes database provided by GraftM developers (package 4.39).

Functional analyses of the metagenomic data were performed with HUMAnN2 v0.6.0 (89). Gene families were grouped by MetaCyc reactions (RXN), keeping unmapped and ungrouped reads for calculating copies per million and relative abundances. MetaCyc RXN groups were filtered, and only those with at least two replicates from the same sampling time point with a relative abundance of 10^−4^ or higher were analyzed (89). MetaCyc RXN notations have been used within Results and Discussion to provide an exact match with the database. RXNs can be considered equivalent to the more traditionally used KO terms from the KEGG database. Grouping by GO terms, specifically performed to evaluate virus presence, indicated values under the threshold levels for all the virus-specific GO terms identified. As such, viruses were not taken in further consideration.

Analysis of variance was performed with the software package STAMP v2.1.3 (90) using the Benjamini-Hochberg false-discovery rate approach (91) for correction of *P* values.

### Accession number(s).

The reads obtained in our study were deposited at the European Nucleotide Archive (PRJEB14718/ERG009353).

## Supplementary Material

Supplemental material
