# Supplementary material for: Response of Microbial Community Function to Fluctuating Geochemical Conditions within a Legacy Radioactive Waste Trench Environment
Source: Appl Environ Microbiol. 2017 Aug 17;83(17):e00729-17. doi: 10.1128/AEM.00729-17 (PMC5561297; doi:10.1128/AEM.00729-17)
Supplement: Supplemental material [file supp_83_17_e00729-17__index.html]

Supplemental material 

# Response of Microbial Community Function to Fluctuating Geochemical Conditions within a Legacy Radioactive Waste Trench Environment

## Supplemental material

- Supplemental file 1 -

  Supplemental results and discussion, daily rainfall at Lucas Heights Meteorological Station from April to July 2015 (Fig. S1), chitobiase (Fig. S2), ISOCIT-CLEAV-RXN (Fig. S3), COENZYME-F420-HYDROGENASE-RXN (Fig. S4), SULFITE-DEHYDROGENASE-RXN (Fig. S5), relevant RXNs related to the nitrogen cycle (Fig. S6), PHOSPHOKETOLASE-RXN and RXN0-310 (Fig. S7), Bray-Curtis similarity tree (Fig. S8), and supplemental file descriptions.

  PDF, 1.2M
