# Supplementary material for: Response of Microbial Community Function to Fluctuating Geochemical Conditions within a Legacy Radioactive Waste Trench Environment
Source: Appl Environ Microbiol. 2017 Aug 17;83(17):e00729-17. doi: 10.1128/AEM.00729-17 (PMC5561297; doi:10.1128/AEM.00729-17)
Supplement: Supplemental material [file AEM.00729-17_zam999118017s1.pdf]

Supplementary Information

for

Response of microbial community function to fluctuating geochemical conditions within a legacy radioactive waste trench environment

Xabier Vázquez-Campos[1,2], Andrew S. Kinsela[2,3], Mark W. Bligh[2,3], Jennifer J. Harrison[3], Timothy E. Payne[3] & T. David Waite[2*]

[1]UNSW Water Research Centre and School of Civil and Environmental Engineering, The University of New South Wales, Sydney, New South Wales 2052, Australia

[2]NSW Systems Biology Initiative, School of Biotechnology and Biomolecular Sciences, The University of New South Wales, Sydney, New South Wales 2052, Australia

[3]Institute for Environmental Research, Australian Nuclear Science and Technology Organisation, Locked Bag 2001, Kirrawee DC, New South Wales 2232, Australia

AEM Journal

*Corresponding author: Professor T.D. Waite; Email: d.waite@unsw.edu.au

Results and Discussion

Reactive oxygen species detoxification

The rapid trench water redox cycling involving the production of large quantities of Fe(II), along with elevated concentrations of organic compounds, creates conditions conducive to reactive oxygen species production (Page *et al.*, 2013; Minella *et al.*, 2015; Klüpfel *et al.*, 2014; Page *et al.*, 2012; Tong *et al.*, 2016). Superoxide dismutase (SOD, SUPEROX-DISMUT-RXN, EC:1.15.1.1) was found to be the predominant RXN related to reactive oxygen species (ROS) detoxification at all time points, with a maximum at day 47 (Figure 5F). Catalase peaked at day 4 and superoxide reductases (SOR, 1.15.1.2-RXN) at day 47, both exhibiting similar relative abundances at the lowest values (days 0 and 4).

The general classic concept of strict anaerobes being unable to cope with O_2 and reactive oxygen species has been long obsolete (Imlay, 2002). While SODs are well distributed amongst aerobic and anaerobic organisms, catalases are more commonly found in aerobes, and SORs in anaerobes (Sheng *et al.*, 2014). This is consistent with the results presented above.

Levels of SOD have been correlated with the aerotolerance of anaerobes (Hassan, 1989; Tally *et al.*, 1977). Gene copy numbers have been correlated with expression levels for numerous proteins. The high relative abundance of SOD during the anaerobic phase could relate to the physiological needs of the anaerobes and aerotolerant microbes thriving in the trenches to deal with transient high oxygen concentrations (Brioukhanov *et al.*, 2002; Sheng *et al.*, 2014).

References

Brioukhanov AL, Thauer RK, Netrusov AI. (2002). Catalase and superoxide dismutase in the cells of strictly anaerobic microorganisms. *Microbiology* **71**: 281–285.

Hassan HM. (1989). Microbial superoxide dismutases. In: Scandalios JG (ed) Vol. 26. *Advances in Genetics*. Academic Press: New York, NY, pp 65–97.

Imlay JA. (2002). How oxygen damages microbes: Oxygen tolerance and obligate anaerobiosis. In: Physiology B-A in M (ed) Advances in Microbial Phisiology Vol. 46. Academic Press, pp 111–153.

Klüpfel L, Piepenbrock A, Kappler A, Sander M. (2014). Humic substances as fully regenerable electron acceptors in recurrently anoxic environments. *Nat Geosci* **7**: 195–200.

Minella M, De Laurentiis E, Maurino V, Minero C, Vione D. (2015). Dark production of hydroxyl radicals by aeration of anoxic lake water. *Sci Total Environ* **527–528**: 322–327.

Page SE, Kling GW, Sander M, Harrold KH, Logan JR, McNeill K, *et al.* (2013). Dark formation of hydroxyl radical in arctic soil and surface waters. *Environ Sci Technol* **47**: 12860–12867.

Page SE, Sander M, Arnold WA, McNeill K. (2012). Hydroxyl radical formation upon oxidation of reduced humic acids by oxygen in the dark. *Environ Sci Technol* **46**: 1590–1597.

Sheng Y, Abreu IA, Cabelli DE, Maroney MJ, Miller A-F, Teixeira M, *et al.* (2014). Superoxide Dismutases and Superoxide Reductases. *Chem Rev* **114**: 3854–3918.

Tally FP, Goldin BR, Jacobus NV, Gorbach SL. (1977). Superoxide dismutase in anaerobic bacteria of clinical significance. *Infect Immun* **16**: 20–25.

Tong M, Yuan S, Ma S, Jin M, Liu D, Cheng D, *et al.* (2016). Production of abundant hydroxyl radicals from oxygenation of subsurface sediments. *Environ Sci Technol* **50**: 214–221.

Supplementary Figures

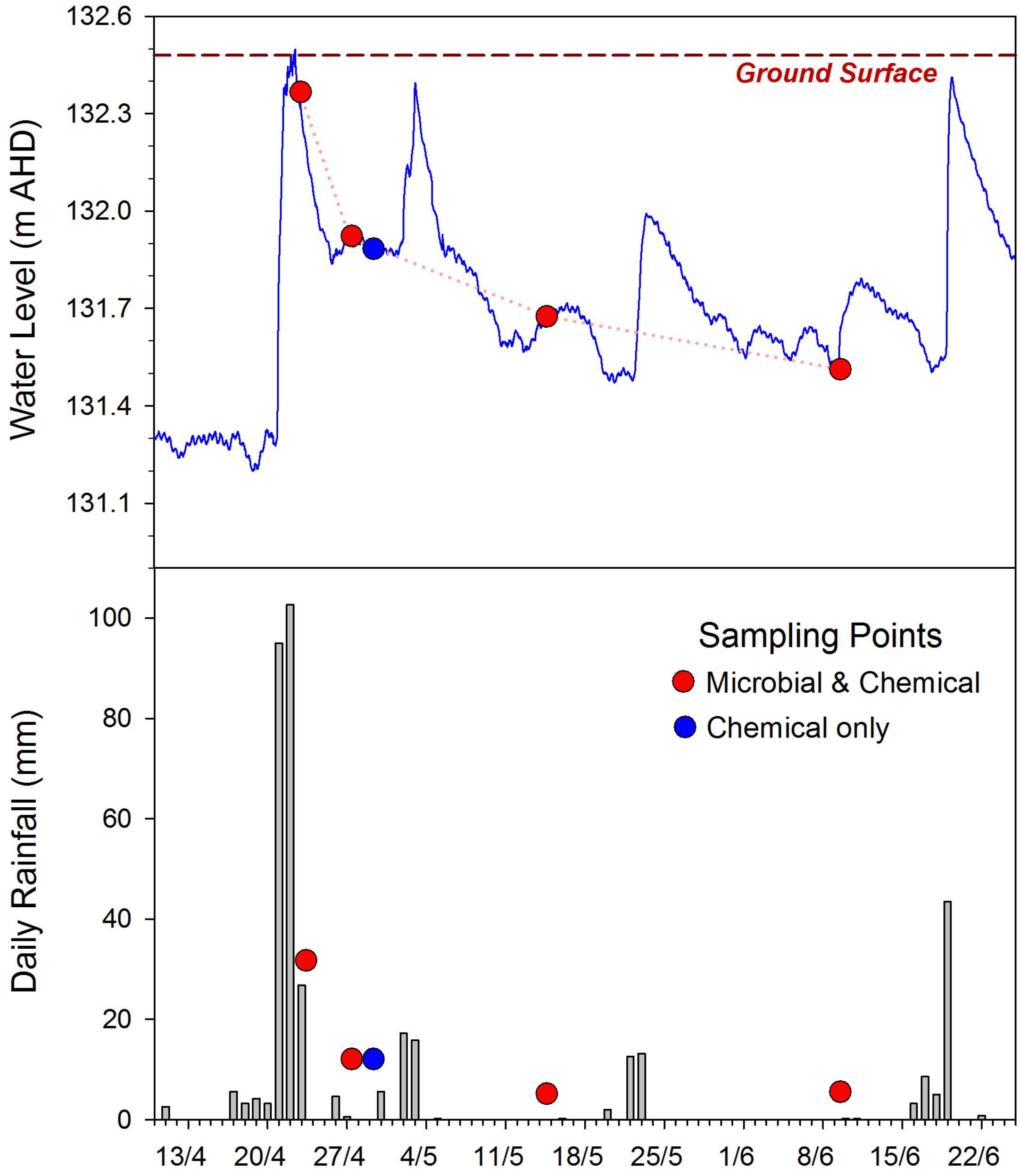

Figure S1. Daily rainfall at Lucas Heights (ANSTO) Meteorological Station from April to July 2015 (bottom). Corresponding trench water levels across the sampling period, along with the ground surface elevation shown by the dashed line (top). Circles depict dates of chemical and microbial sampling. Rainfall data is courtesy of the Australian Government, Bureau of Meteorology.

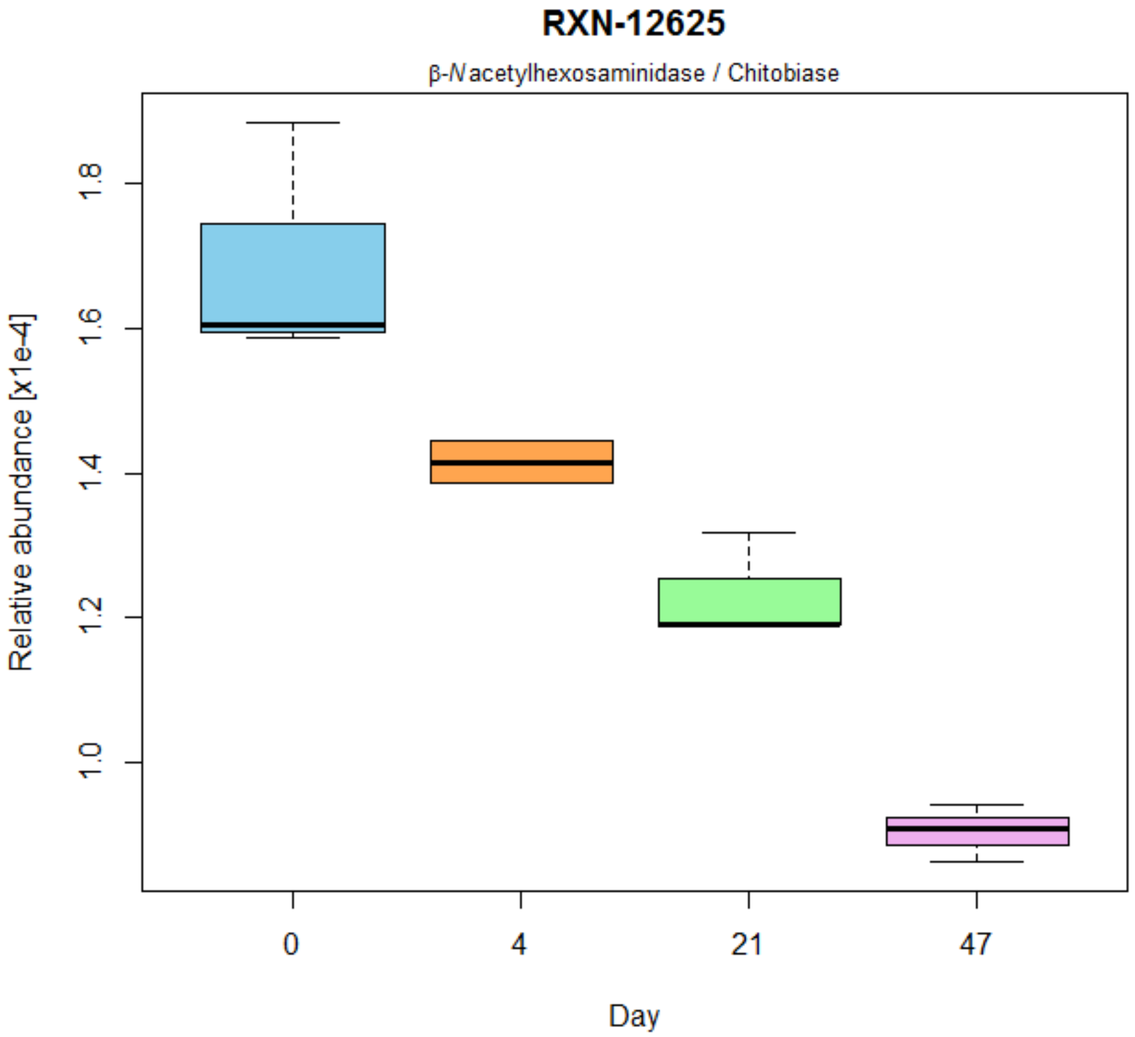

Figure S2. Chitobiase. Marker for the degradation of chitobiose and/or chitin.

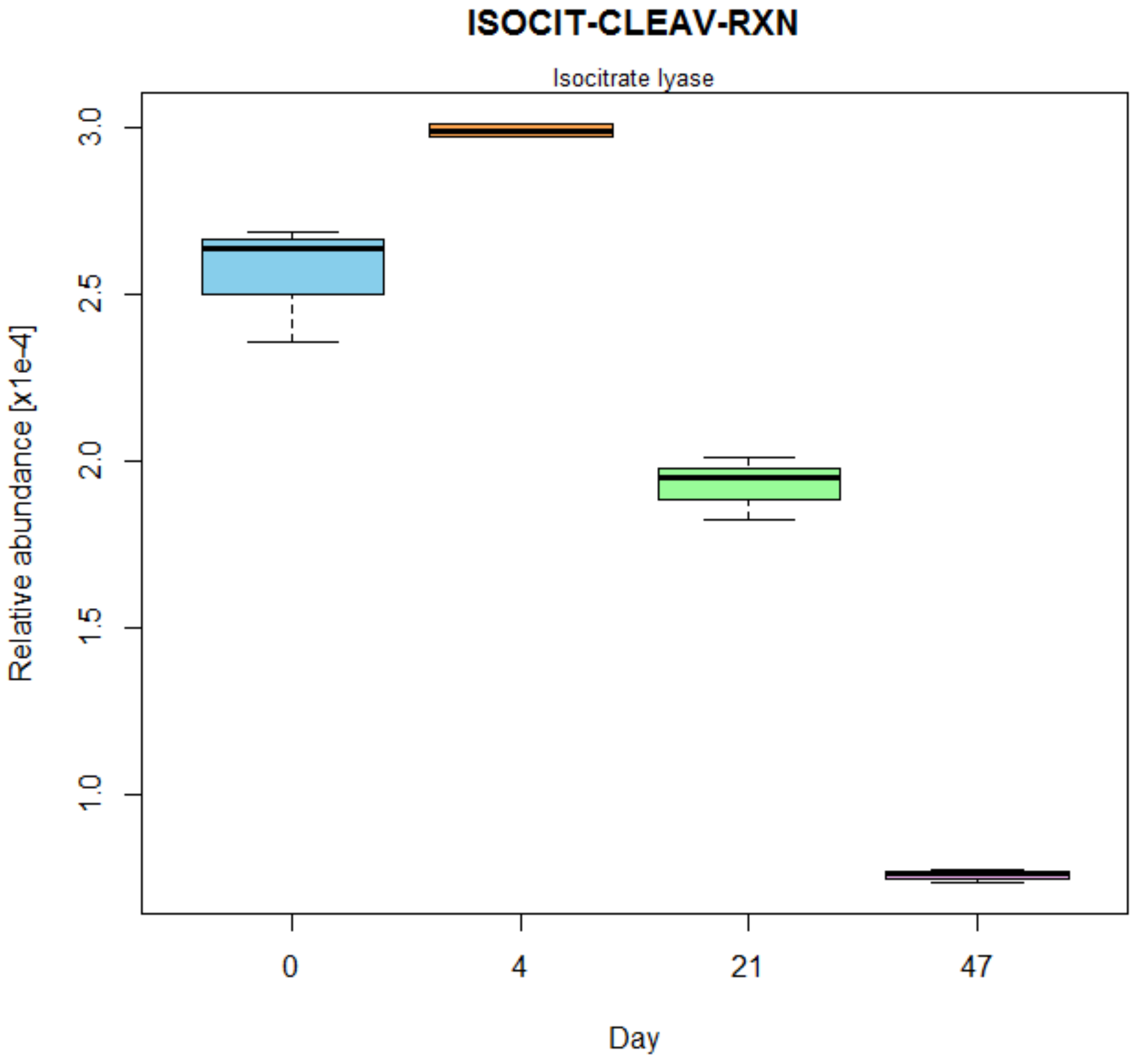

75
76 **Figure S3. ISOCIT-CLEAV-RXN.** Marker for the glyoxylate pathway.

77

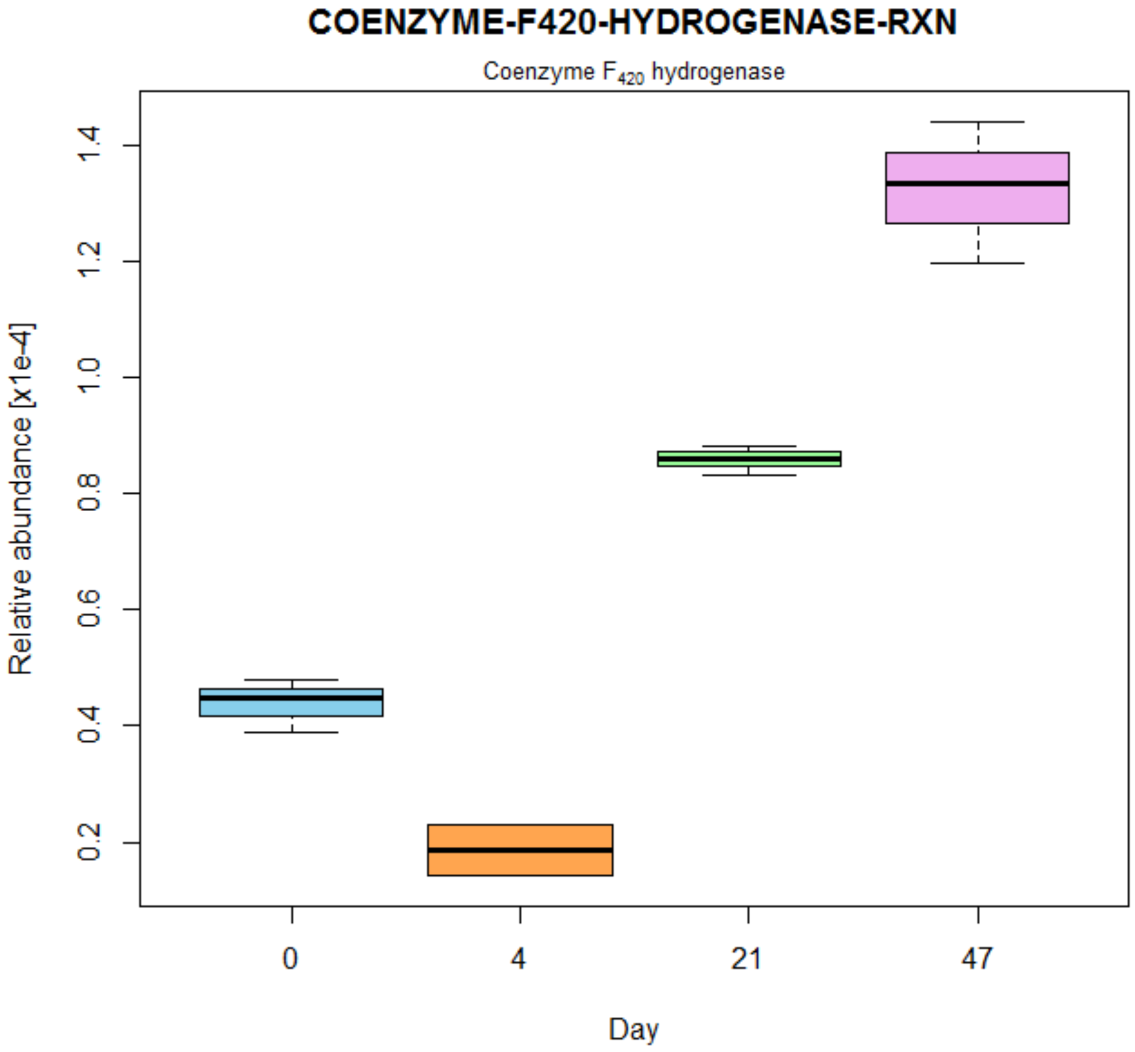

Figure S4. COENZYME-F420-HYDROGENASE-RXN. Indicator of methanogenesis from H_2 and CO_2.

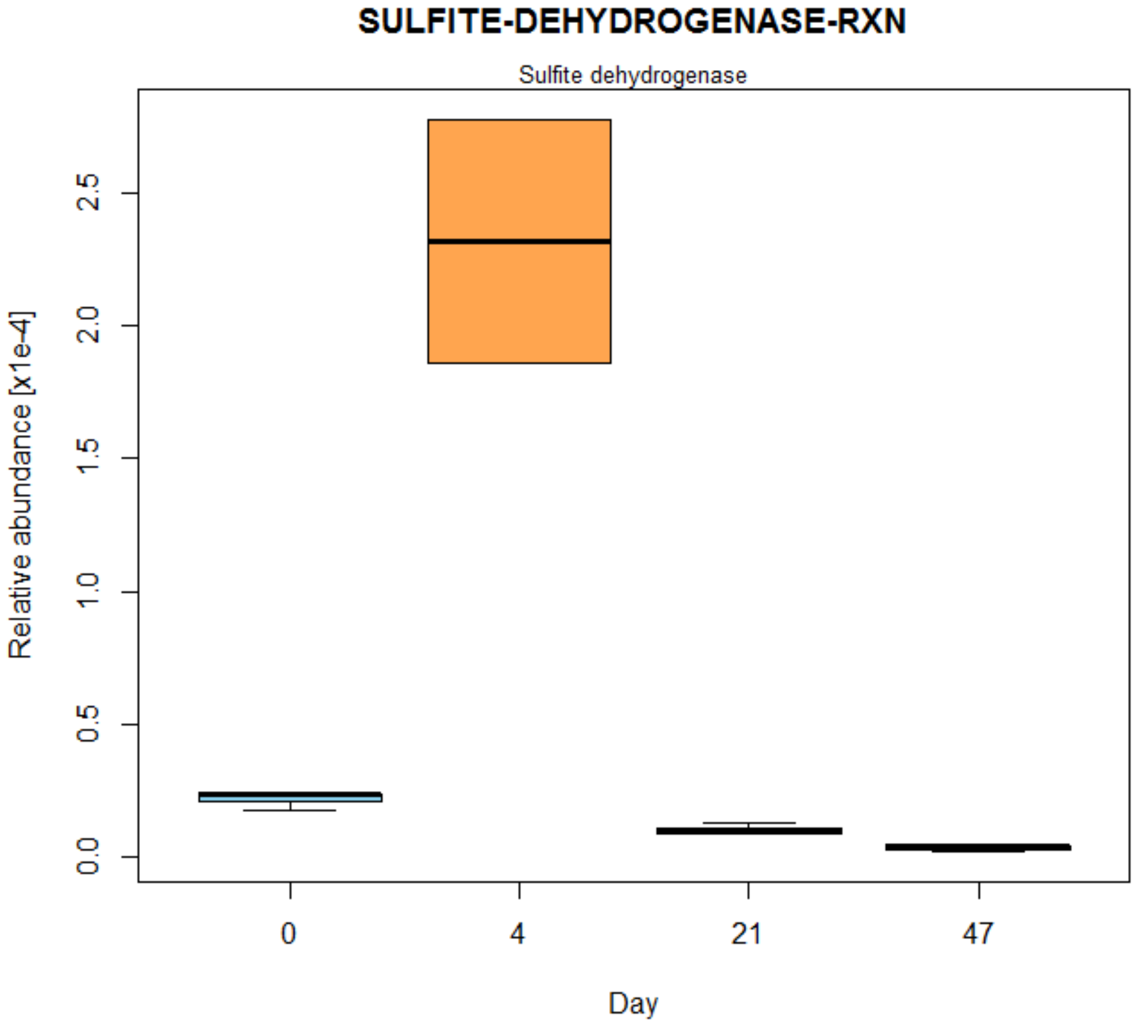

Figure S5. SULFITE-DEHYDROGENASE-RXN (EC:1.8.2.1). Assimilatory sulfate reduction.

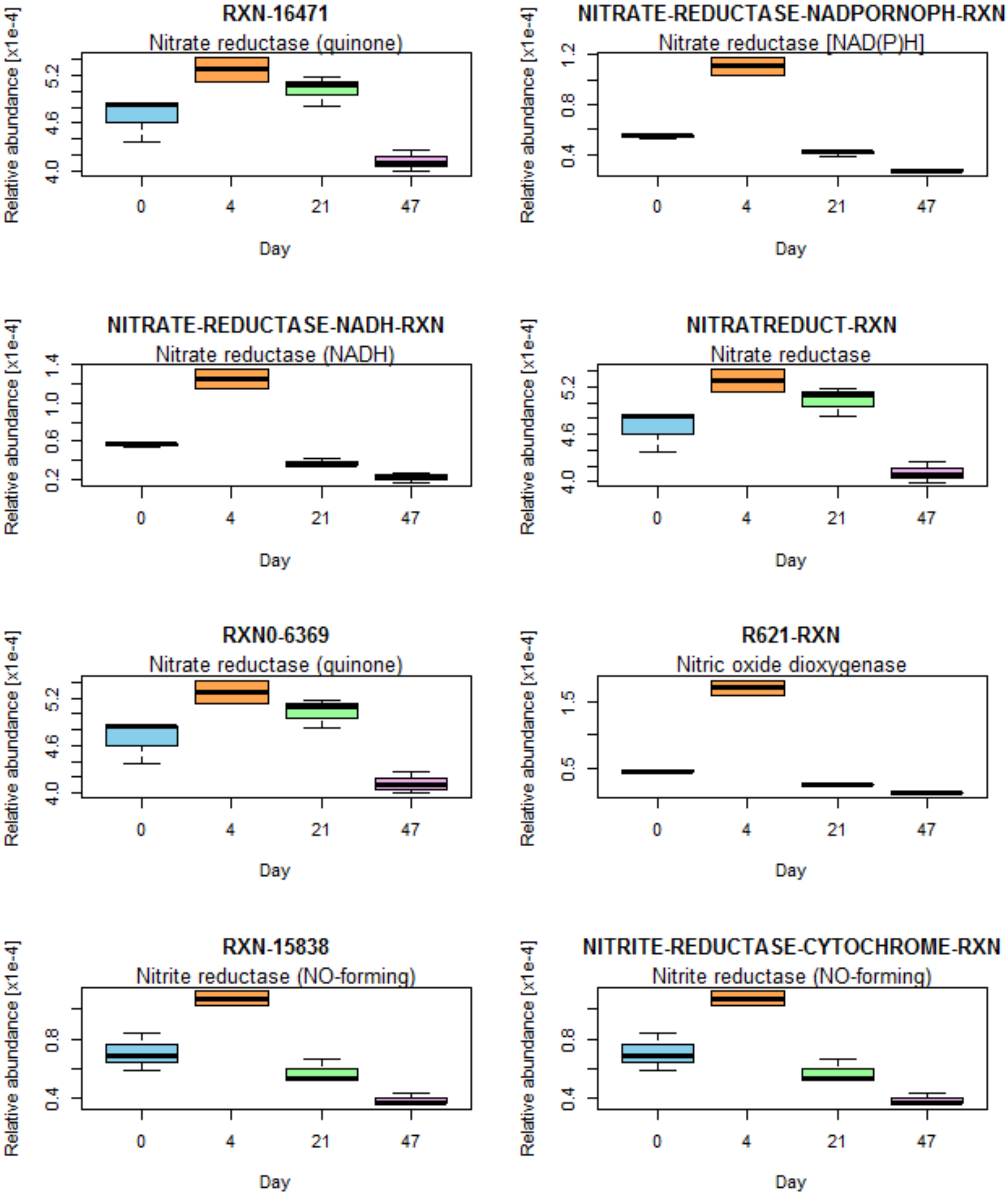
RXN-16471
Nitrate reductase (quinone)
NITRATE-REDUCTASE-NADPORNOPH-RXN
Nitrate reductase [NAD(P)H]
NITRATE-REDUCTASE-NADH-RXN
Nitrate reductase (NADH)
NITRATREDUCT-RXN
Nitrate reductase
RXN0-6369
Nitrate reductase (quinone)
R621-RXN
Nitric oxide dioxygenase
RXN-15838
Nitrite reductase (NO-forming)
NITRITE-REDUCTASE-CYTOCHROME-RXN
Nitrite reductase (NO-forming)
Relative abundance [x1e-4]
Day
0
4
21
47

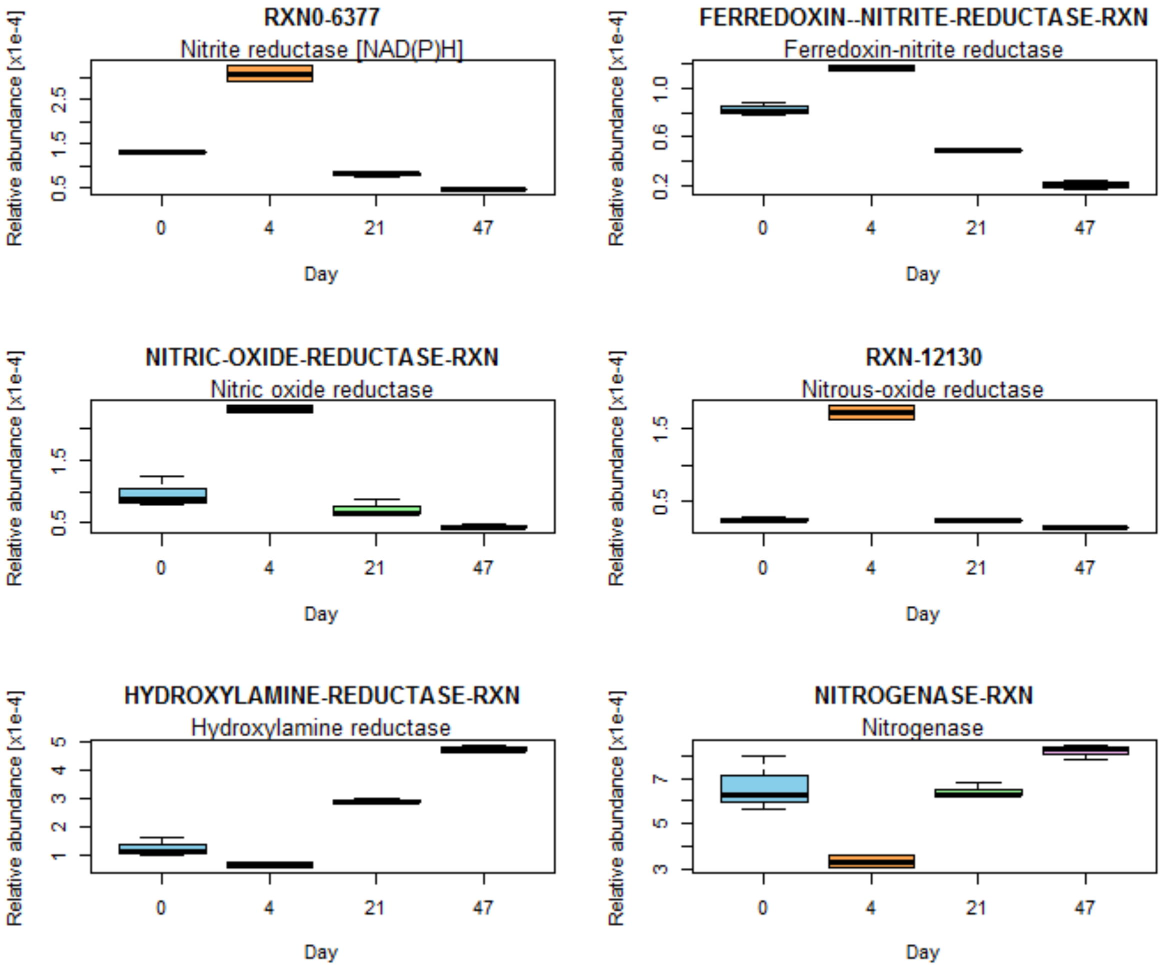

Figure S6. Relevant RXNs related to the nitrogen cycle.

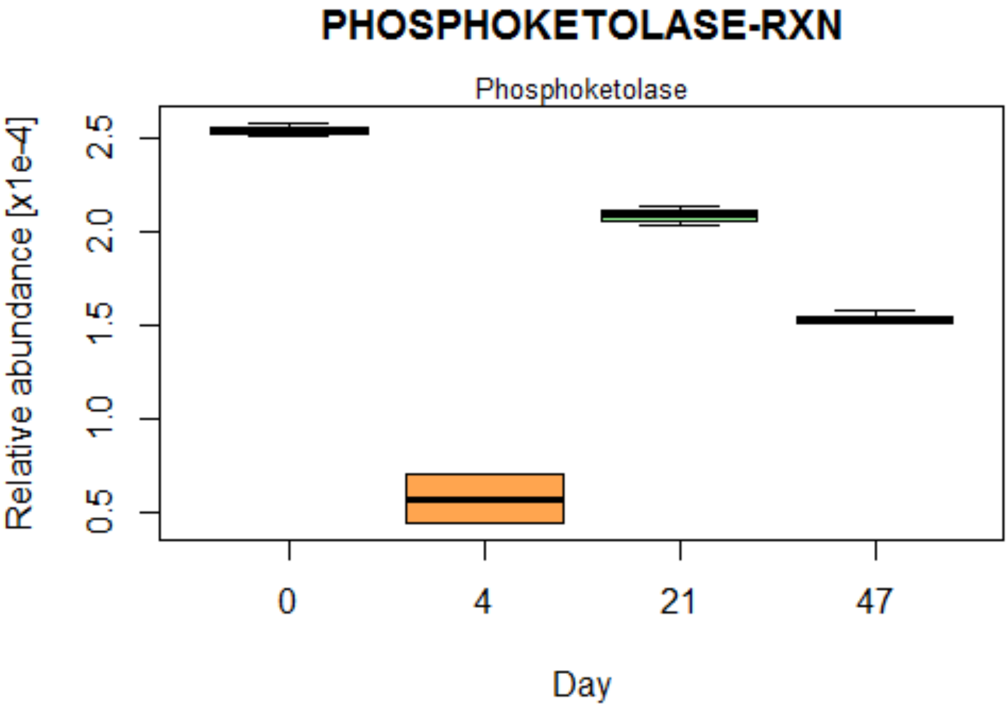

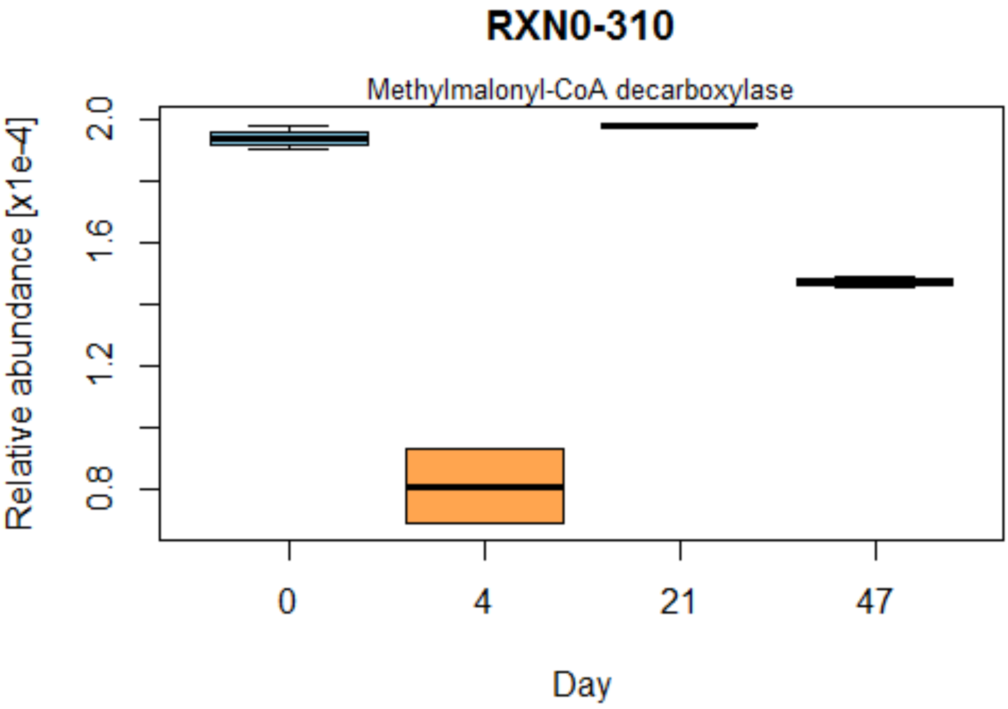

Figure S7. PHOSPHOKETOLASE-RXN and RXN0-310. Markers for heterolactic and propionate fermentation respectively.

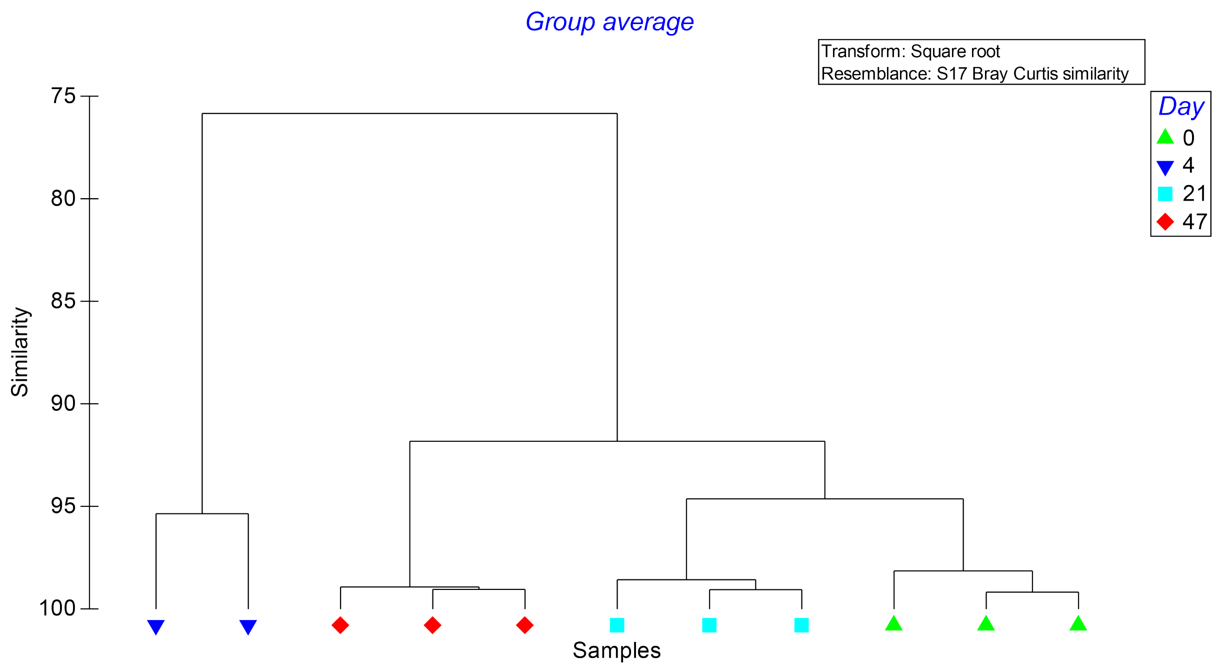

Figure S8. Bray-Curtis similarity tree of the functional profiles for the individual sampling replicates.

Supplementary Files

Supplementary file 1. Spreadsheet with YSI data.

Supplementary file 2. Krona HTML file with the taxonomy.

Supplementary file 3. Spreadsheet with the relative abundances of all the RXNs.

Supplementary file 4. Table with the raw HUMAnN2 output.

All supplementary files can be viewed at: https://dx.doi.org/10.6084/m9.figshare.3817356
